# Colony stimulating factors secreted by irradiated autologous tumor cell vaccines inhibit immunity

**DOI:** 10.1186/2051-1426-3-S2-P448

**Published:** 2015-11-04

**Authors:** Sruthi Ravindranathan, Sean G Smith, Khue Nguyen, David A Zaharoff

**Affiliations:** 1 University of Arkansas, Fayetteville, AR, USA

## 

Autologous tumor cell-based vaccines (ATCVs) have a number of potential advantages including multivalency and patient specificity. ATCVs contain many potential antigens, both known and unknown which potentiate polyclonal responses capable of responding to a more diverse population of tumor cells. In addition, because ATCVs are created from a patient's own tumor, all potential immunogenic epitopes are patient specific and each patient is immunized against her complete and individualized antigen repertoire. This is particularly important for breast cancer as each tumor can contain up to 100 different mutant genes so no two tumors are identical.

The major disadvantage of ATCVs, is poor immunogenicity. In order to develop an effective, immunogenic ATCV against breast cancer, we wanted to understand the features of immunogenicity. In this study, BALB/c female mice were given priming and booster vaccinations, ten days apart, with 1 million irradiated (100Gy) EMT6 and/or 4T1cells. Ten days after the booster vaccination, mice were challenged with live tumor cells. 80% of mice vaccinated with EMT6 cells were completely protected against a live EMT6 challenge. However, mice vaccinated with irradiated 4T1 cells failed to provide any protection against a live 4T1 challenge. Most interestingly, when mice were vaccinated with a mixture of irradiated EMT6 and 4T1 cells at the same site, the protective response against EMT6 challenge was significantly diminished as 60% of mice developed tumors. Furthermore, when irradiated EMT6 and 4T1 cells were administered on opposite sides, protection from an EMT6 challenge was also significantly diminished with 88% of mice developing tumors. This finding implied that non-immunogenic irradiated 4T1 cells released one or more immunosuppressive factors that inhibited anti-EMT6 immunity.

Thus, we investigated the levels of different immunosuppressive cytokines, G-CSF, M-CSF, GM-CSF, IL-6, MCP-1, TGF-β and VEGF released by both 4T1 and EMT6 cells before and after irradiation. Irradiated 4T1 cells secreted high levels of colony stimulating factors. Specifically, at 24 and 48 hours after irradiation, 4T1 cells secreted 60 and 705 pg/ml of M-CSF; 912 and 5190 pg/ml of G-CSF; 29 and 180 pg/ml of GM-CSF. We believe that high levels of colony stimulating factors induce the accumulation of large amounts of myeloid derived suppressor cells (MDSCs) in the tumor site and lymphoid organs which, in turn, suppress anti-EMT-6 immunity. Future studies will determine if blocking colony stimulating factors will decrease the accumulation of MDSCs and subsequently increase anti-tumor immunity.

